# Serum golgi protein-73 (GP-73) in children with autoimmune hepatitis

**DOI:** 10.1007/s00431-025-06428-7

**Published:** 2025-09-03

**Authors:** Marwa Sabry Rizk, Ola Galal Ali Behairy, Amera Mohamed Abdel Salam Dawood, Rana Atef Khashaba, Walaa El Gendy, Nashwa Farouk Mohamed

**Affiliations:** 1 Pediatric Hepatology, Gastroenterology and Nutrition Department, National Liver Institute, Menoufia University, Menoufia, Egypt; 2 Pediatrics Department, Faculty of Medicine, Benha University, Benha, Egypt; 3 Pediatrics Department, Faculty of Medicine, Tanta University, Tanta, 31511 Egypt; 4 Clinical Pathology and Chemistry Department, Faculty of Medicine, Benha University, Benha, Egypt; 5 Pathology Department, National Liver Institute, Menoufia University, Menoufia, Egypt

**Keywords:** Autoimmune hepatitis, Liver fibrosis, Children, Serum golgi protein-73

## Abstract

To assess the correlation between serum Golgi protein-73 (GP-73) and the degree of hepatic fibrosis in children with autoimmune liver diseases. This case–control study included 100 children. Group A involved 50 children with autoimmune hepatitis (AIH), aged 2–17 years (mean 11.3 ± 2.7 years), while Group B included 50 age- and sex-matched healthy controls. All participants underwent clinical assessment, abdominal ultrasonography, laboratory investigations, and measurement of serum GP-73 using enzyme-linked immunosorbent assay. Children with AIH had significantly higher serum GP-73 levels than controls (median 110.7 ng/L vs. 21.3 ng/L, *p* < 0.001). GP-73 levels showed a strong positive correlation with both the histological activity index (r = 0.879, *p* < 0.001) and the stage of fibrosis (r = 0.647, p < 0.001). At a cutoff level > 49 ng/L, GP-73 distinguished AIH cases with 100% sensitivity and 100% specificity. For identifying severe fibrosis, a cutoff > 124.6 ng/L yielded 100% sensitivity and 91.7% specificity (area under the ROC curve = 0.995, *p* < 0.001).

*Conclusion*: Serum GP-73 levels are significantly elevated in children with autoimmune hepatitis and correlate strongly with both histological grading and staging. GP-73 may serve as a reliable non-invasive marker for assessing disease severity and guiding clinical management in pediatric autoimmune liver diseases.

**Table:** 

**What is Known:** • *Non-invasive biomarkers for assessing liver fibrosis in children with autoimmune hepatitis are still limited, and liver biopsy remains the gold standard despite its risks.*
**What is New:** • *Serum GP-73 is significantly elevated in pediatric AIH, strongly correlates with fibrosis severity, and may serve as a reliable non-invasive biomarker.*

**Supplementary Information:**

The online version contains supplementary material available at 10.1007/s00431-025-06428-7.

## Introduction

Autoimmune liver diseases (ALDs) encompass a broad range of illnesses with common clinical and pathological features. The two primary forms by which these diseases are classified are autoimmune hepatitis (AIH) and autoimmune sclerosing cholangitis. The main definition for this category is when cholangiography shows involvement of the bile ducts. AIH is further classified into type 1 (AIH-1) and type 2 (AIH-2), based on the autoantibody profile [1].

Serum bilirubin levels rise, albumin levels fall, and the international normalized ratio (INR) remains elevated as a result of AIH’s disruption of the liver’s excretory and synthetic processes [2]. In the largest, mostly European cohorts, the potential modes of AIH manifestation include: sudden onset, resembling viral hepatitis, with non-specific symptoms such as fatigue, vomiting, anorexia, joint and abdominal pain, followed by dark urine, pale stool, and jaundice; severe hepatic failure with hepatic encephalopathy (grades II to IV); or gradual onset, with vague symptoms lasting for six months to several years before diagnosis [3].

Diagnosing cirrhosis and liver fibrosis is clinically important for patients with chronic liver disease because it allows for the prediction of complications, optimization of management, and the design of a surveillance strategy. One important indicator of prognosis in this group is liver fibrosis [4].

Patients suspected of having AIH should undergo a liver biopsy to confirm the diagnosis, assess the stage of fibrosis, and determine the best course of treatment. Liver biopsies are the diagnostic gold standard for AIH patients, but they come with a number of drawbacks, such as the need for highly trained medical professionals, large variations in results between observers, and the possibility of serious complications during and after the procedure. The use of repeated liver biopsies to monitor a patient’s response to therapy over an extended period of time is inappropriate. In order to make treatment decisions and accurate prognosis estimations, non-invasive measurements are required to detect and stage fibrosis in these patients [5].

A transmembrane protein known as Golgi protein-73 (GP-73) has been discovered on the Golgi apparatus. Golgi membrane protein I or Golgi phosphoprotein 2 is another name for it. It is also known as GP-73 due to its relative molecular mass of 7.3 × 10^1^ in polyacrylamide gel electrophoresis. The portal areas of bile duct epithelial cells are the primary sites of its expression. Although normal liver cells express very little, this location is typical of the Golgi apparatus in epithelial cells [6]. Serum from patients with liver diseases contains GP-73, and epithelial cells in numerous human tissues express this protein. Despite the available evidence on GP-73 in adult populations, its diagnostic utility in assessing liver fibrosis among children with autoimmune hepatitis (AIH) remains insufficiently explored. This study seeks to fill this important knowledge gap. Therefore, this study investigates whether serum GP-73 can serve as a reliable non-invasive biomarker for assessing the severity of liver fibrosis in children with autoimmune hepatitis [7]. This study aims to provide a non-invasive alternative for liver biopsy by evaluating serum GP-73 levels in relation to fibrosis severity in pediatric AIH patients.

## Patients and methods

This case–control study was conducted on 100 children as follows: Group A included 50 children who attended the Pediatric Hepatology Unit at Benha University Hospitals and the National Liver Institute, Menoufia University. The laboratory work was performed in the Clinical Pathology Department, Benha University, between December 2022 and April 2024. Their ages ranged from 2 to 17 years, with 24 males and 26 females. The mean age was 11.3 ± 2.7 years.

Group B included 50 apparently healthy children, matched for age and sex, who were recruited as a control group from the general pediatric outpatient clinics during routine health visits. These children had no history of liver disease, autoimmune disorders, or chronic illness. All controls underwent clinical evaluation, abdominal ultrasonography, and basic laboratory investigations, including liver function tests (ALT, AST, bilirubin, and albumin), to confirm the absence of subclinical liver disease. Only those with completely normal findings were included in the control group.

Informed written consent was obtained from the parents or guardians of all participants after they were fully informed about the study.

As for the AIH group, inclusion criteria included children under 18 years of age with a confirmed diagnosis of autoimmune hepatitis based on the revised criteria of the International Autoimmune Hepatitis Group (IAIHG), as recommended by the updated EASL guidelines (2025) [8] including serological markers (e.g., ANA, ASMA, LKM-1), elevated immunoglobulin G (IgG), liver function abnormalities, and histological confirmation via liver biopsy. All patients were treatment-naïve at the time of liver biopsy.

Patients subsequently received immunosuppressive therapy after diagnosis and during the period of serum sample collection. Exclusion criteria included children with other chronic liver diseases (e.g., viral hepatitis B or C, metabolic liver disease, cholestatic conditions),as recommended by EASL, 2025 in addition to those with systemic comorbidities (renal, cardiac, or neurological), and patients with incomplete diagnostic data.

Thorough history taking was conducted on all patients enrolled in the study. This included a comprehensive assessment of the medical background covering age, sex, presenting complaint, and the first manifestation of the disease. Past medical history included information such as blood transfusions, HBV vaccination, use of hepatotoxic drugs, and other autoimmune diseases. Illness duration and follow-up duration were also recorded. Each patient was asked about abnormalities during the neonatal period or prior to the current illness, as well as the onset and current symptoms of the disease, and any other medical issues or treatments. Family history included information about parental consanguinity, shared medical conditions, and a history of autoimmune diseases in first-degree relatives.

All patients underwent a complete physical examination. Anthropometric measurements included body mass index (BMI), height, and weight. Percentiles were calculated using pediatric reference charts from Egypt. Abdominal examination included inspection, percussion, and both superficial and deep palpation to assess the liver and spleen.

### Abdominal ultrasonography

(US) was performed to assess liver size and texture, spleen size, and presence of ascites, using normal reference ranges.

### Laboratory investigations

Included complete blood count and liver function tests: transaminases (AST, ALT), alkaline phosphatase, gamma-glutamyl transpeptidase, total and direct bilirubin, and serum albumin. Serum immunoglobulin G (IgG) levels and autoantibodies were also measured, including quantitative determination of antinuclear antibodies (ANA), smooth muscle antibodies (ASMA), liver-kidney microsomal antibodies (LKM-1), and antimitochondrial antibodies (AMA).

Serum GP-73 levels were measured using a human GP-73 ELISA kit (Bioassay Technology Laboratory, China, Cat. No. E1432Hu). The assay detection range was 0.3–290 ng/mL, with intra-assay and inter-assay coefficients of variation < 8% and < 10%, respectively. The reference range for healthy children in this study was 21.3 ± 2.5 ng/mL, based on the control group data.

### Liver biopsy (AIH group only)

US-guided liver biopsy was performed in all patients using a fine needle aspiration (Mangini needle) for cases from the Pediatric Gastroenterology and Hepatology Unit at Benha University and a true cut needle for cases from the National Liver Institute. A sufficient liver tissue core was defined as the presence of eleven or more portal tracts. Biopsy specimens were fixed in formalin, embedded in paraffin, sectioned at 5 μm thickness, and stained with hematoxylin and eosin, Masson’s trichrome, and Perls’Prussian blue for fibrosis evaluation. PAS stain was used to exclude alpha-1-antitrypsin deficiency.

Histological evaluation included the histological activity index (HAI) and fibrosis staging. HAI ranged from 0 to 18:1–3: minimal,4–8: mild,9–1: moderate,13–18: severe.

Fibrosis was scored on a scale from 0 to 6. The criteria included presence of portal-to-portal bridging, nodules, incomplete cirrhosis, and fibrous expansion of portal areas. Cirrhosis was diagnosed based on architectural distortion [9]. Representative histopathological images of liver biopsies, illustrating different stages of fibrosis and interface hepatitis, are provided in the supplementary file (see Supplementary Figs. 1, 2, 3, 4, 5, 6, 7 and 8).

Prognostic scoring systems used included:

### Child–Pugh score

Assesses severity of liver dysfunction based on ascites, neurological status, nutritional state, and serum albumin and bilirubin levels [10].

### PELD score (for children < 12 years)

PELD = 0.480 [Ln serum bilirubin (mg/dL)] + 1.857 [Ln INR] – 0.687 [Ln albumin (g/dL)] + 0.436 (if age < 1 year old) + 0.667 (if growth failure present) [11].

### MELD score (for children ≥ 12 years)

MELD = 3.78 [Ln bilirubin (mg/dL)] + 11.2 [Ln INR] + 9.57 [Ln creatinine (mg/dL)] + 6.43 [12]. Based on the age distribution (3–17 years, mean ≈11 years), PELD was applied to children < 12 years and MELD to those ≥ 12 years, in line with pediatric hepatology guidelines.

### APRI score

Calculated as (AST/ULN) × 100/PLT (10⁹/L), used to estimate severity of liver fibrosis [13].

FIB-4 index: FIB-4 = Age (years) × AST (U/L)/[PLT (10⁹/L) × √ALT (U/L)] [14]**.**

### Sample size

Sample size was calculated using Cochran’s formula:

$$n\:=\:Z^2\:\times\:p\:\times\:q/e2^{}$$

Where Z = 1.96 for 95% confidence level, p = estimated prevalence (0.15), q = 1 – p (0.85), and e = margin of error (0.1).

Based on Hahn et al. [15], the global prevalence of autoimmune hepatitis is approximately 15%, resulting in a required sample size of ~ 50 per group:

n ≈ (1.96)^2^ × 0.15 × 0.85/(0.1)^2^ ≈ 50 patients per group.

### Ethical considerations

The authors assert that all procedures contributing to this work adhere to the ethical standards of the relevant guidelines of declaration of Helsinki and have been approved by the appropriate committees at our institution, Research Ethics Committee Faculty of Medicine (RECFM),Benha University (No.Ms.50.5.2023).Written consents were obtained from parents/guardians after being fully informed about the nature of the study and its procedures.

#### Statistical analysis

We used SPSS v27 (IBM©, Armonk, NY, USA) for our statistical analysis. To determine if the data was normally distributed, the Shapiro-Wilks test and histograms were employed. An unpaired student t-test was used to analyze the quantitative parametric data, and the results showed a mean and standard deviation (SD). we used non parametric tests due to the non-normal distribution of most variables. For quantitative non-parametric data, the Mann Whitney U-test was employed to determine the median and interquartile range (IQR). We utilized Fisher’s exact test or a Chi-square test as needed, and for qualitative variables, we expressed the outcomes as percentages and frequencies. A two-tailed P value below 0.05 was considered statistically significant. We used the Pearson or Spearman correlation to estimate the degree of correlation between two quantitative variables. All tests were evaluated for their diagnostic performance using ROC curve analysis, and diagnostic sensitivity, specificity, PPV, and NPV were used for that purpose. Using ROC curve analysis, we determined how well each test performed in terms of overall diagnostic accuracy.

Effect Size Calculation:

Effect Size (r) for Mann–Whitney U Test as recommended by Field [16].

Z-score: 5.

Total sample size (N): 100 (50 AIH + 50 Controls).

Formula:

$$r\:=\:\frac Z{\sqrt N}\:=\:\frac{5.0}{\sqrt{100}}\:=\:\frac{5.0}{10}\:=\:0.50$$

Interpretation:

Effect size (r) ≈ 0.50. This indicates a large effect size, meaning the difference in GP-73 levels between the AIH and control groups is not only statistically significant but also clinically meaningful. Although multiple comparisons were conducted, no formal correction was applied. Therefore, p-values should be interpreted with caution.

## Results

There was no statistically significant difference between AIH group and controls regarding age, sex or.

consanguinity. Patients in AIH group had statistically significant higher frequency of other autoimmune.

disease (34% vs 4%, p < 0.001). Regarding anthropometric measurements and presenting symptoms, AIH patients had statistically significantly lower weight, height and BMI percentiles.

compared to healthy controls. most patients (72%) had jaundice, 70% had abdominal pain, 44% had abdominal enlargement and 40% had pallor, while none of our cases had gastrointestinal bleeding. **(**Table 1**).**

**Table 1:** Baseline anthropometric characteristics and clinical presentation of AIH patients compared to controls

		AIH group *N* = 50 (%)	Control group *N* = 50 (%)	Test	*P* value
Age (years)		11.30 ± 2.70	10.50 ± 3.10	t = 0.32	0.72
Sex	Male	24 (48.00%)	25 (50.00%)	X2 = 0.36	0.54
	Female	26 (52.00%)	25 (50.00%)		
Consanguinity		20 (40.00%)	14 (28.00%)	X2 = 1.60	0.20
Other autoimmune diseases	DM	10 (20.00%)	2 (4.00%)	X2 = 15.10	0.002*
	IBD	2 (4.00%)	0 (0.00%)		
	Thyroid	1 (2.00%)	0 (0.00%)		
	Vitiligo	1 (2.00%)	0 (0.00%)		
	RA	3 (6.00%)	0 (0.00%)		
Anthropometric measurements	Weight (kg)	29.50 ± 14.10	37.20 ± 12.10	t = 2.60	0.012*
	Weight (percentile)	38.60 ± 25.40	46.50 ± 13.30	t = 2.90	0.007*
	Height (cm)	129.30 ± 13.40	142.60 ± 16.20	t = 3.10	0.002*
	Height (percentile)	45.50 ± 16.50	52.90 ± 18.90	t = 2.40	0.020*
	BMI (kg/m^2^)	16.50 ± 3.10	18.90 ± 4.30	t = 3.10	0.003*
	BMI (percentile)	31.20 ± 13.40	51.20 ± 19.30	t = 3.60	< 0.001*
Clinical presentation	Abdominal enlargement	36 (72.00%)	––	––	––
	Pallor	36 (72.00%)	––	––	––
	Jaundice	22 (44.00%)	––	––	––
	Abdominal pain	20 (40.00%)	––	––	––

Data are presented as mean ± SD or frequency (%). *t,* Student t-test; *X*^*2*^, Chi square test; *DM*, diabetes mellitus; *IBD*, inflammatory bowel disease; *RA,* rheumatoid arthritis; *BMI,* body mass index, *: significant as P value ≤ 0.05

Laboratory investigations revealed significantly elevated levels of WBCs, ALT, AST, GGT, ALP, total and direct bilirubin, PT, PTT, INR, and total IgG in the AIH group. In contrast, hemoglobin, platelet count, and albumin levels were significantly lower in these patients. Additionally, they had a higher incidence of ascites and organomegaly, along with increased liver and spleen sizes. Serum GP-73 levels were markedly elevated in AIH patients compared to controls **(**Table 2**).**

**Table 2:** Comparison of laboratory investigations, abdominal ultrasound findings,and Golgi Protein73 levels between children with autoimmune hepatitis(AIH) and healthy controls

			AIH group (*N* = 50)	Control group (*N* = 50)	Test	*P* value
Laboratory investigations	Hemoglobin (g/L)		10.10 ± 1.50	11.70 ± 1.10	*t* = 5.90	< 0.001*
	WBCs (× 10^3^/µl)		8.10 ± 30	6.50 ± 1.90	*t* = 2.90	0.003*
	Platelets (× 10^3^/µl)		148 ± 56	323 ± 61	*t* = 14.90	< 0.001*
	ALT (U/L)		200 (170–313)	33 (32–35)	*U* = 6.90	< 0.001*
	AST (U/L)		298 (236–353)	33 (30–44)	*U* = 10 20	< 0.001*
	GGT (U/L)		34 (30–40)	24 (23–27)	*U* = 4.10	< 0.001*
	Alkaline phosphatase (U/L)		139 (125–165)	98 (92–108)	*U* = 4.20	< 0.001*
	Bilirubin Total (mg/dl)		1.10 (0.90–2)	0.70 (0.50–0.90)	*U* = 4.60	< 0.001*
	Bilirubin direct (mg/dl)		0.40 (0.30–1.60)	0.20 (0.20–0.30)	*U* = 4.50	< 0.001*
	Albumin (g/dL)		3.50 ± 0.70	4.40 ± 0.50	*t* = 7.60	< 0.001*
	aPTT (sec)		50 ± 13	35 ± 4	*t* = 7.40	< 0.001*
	PT (sec)		17.50 ± 5.90	12 ± 0.70	*t* = 5.50	< 0.001*
	INR		1.50 ± 0.40	1.10 ± 0.10	*t* = 5.40	< 0.001*
	Total IgG (mg/dl)	Median	2399	813	*U* = 10 20	< 0.001*
		IQR	2083–2570	765–716		
	Creatinine (mg/dl)		0.90 ± 0.20	0.80 ± 0.10	*t* = 0.90	0.31
Abdominal ultrasound	Organomegaly	Hepatomegaly	11 (22.00%)	0 (0.00%)	*X*^2^ = 27 .30	< 0.001*
		Splenomegaly	5 (10.00%)	0 (0.00%)		
		HSM	26 (52.00%)	0 (0.00%)		
	Ascites		12 (24.00%)	0 (0.00%)	*X*^2^ = 3 90	0.003*
	Liver span (cm)		13.10 ± 1.20	11.70 ± 1.10	*t* = 5.50	< 0.001*
	Spleen size (cm)		12.30 ± 1.60	10.40 ± 1.30	*t* = 6.70	< 0.001*
GP-73 (ng/L)			110.70 ± 26.50	21.30 ± 2.50	*U* = 8.60	< 0.001*

Data are presented as mean ± SD, median (IQR), or frequency (%). *t*, Student’s t-test; *U,* Mann–Whitney U test; *χ*^2^: Chi-square test; *Hb,* hemoglobin; *WBCs,* white blood cells; *ALT,* alanine aminotransferase; *AST,* aspartate aminotransferase; *GGT,* gamma-glutamyl transferase; *PT,* prothrombin time; *aPTT,* activated partial thromboplastin time; *INR,* international normalized ratio; *IgG,* immunoglobulin G; *GP-73,* Golgi protein 73; *AIH,* autoimmune hepatitis. Statistically significant at *p* ≤ 0.05

Autoantibody profiles, liver biopsy scores, treatment protocols, and patient responses were documented within the AIH group to provide insight into disease characterization and management. ANA was negative in 56% of patients, 1/40 in 22% of patients, 1/80 in 20% of patients ASMA was negative in 18%, 1/40 in 30% of patients, 1/80 in 48%, and > 1/80 in 4% of patients. Anti-LKM1 was negative in 92%, 1/80 in 4% of patients and > 1/80 in 4% of patients. AMA was negative in 90% and positive in 10% of patients. Most cases (82%) were of type I AIH. In the present study, the mean revised original scoring system in the studied group was 22.1 ± 3.2, the mean simplified scoring system was 6.9 ± 0.7, the mean Child–Pugh score was 7.9 ± 1.6, the mean PELD was 8.5 ± 3.5,the mean MELD was 9.4 ± 3.2, the mean APRI score was 5.0 ± 3.1 and the mean FIB-4 score was 0.9 ± 0.3. All cases received steroids, then 52% received Steroid and Azathioprine. 58% of cases had remission and 42% had relapse **(**Table 3**).**

**Table 3:** Autoantibody profiles, liver histopathology, diagnostic scores, treatment regimens, and treatment response among children with autoimmune hepatitis (AIH)

				AIH group *N* = 50 (%)
ANA		Negative < 1/40		28 (56.00%)
		1/40		11 (22.00%)
		1/80		10 (20.00%)
ASMA		Negative < 1/40		9 (18.00%)
		1/40		15 (30.00%)
		1/80		24 (48.00%)
		> 1/80		2 (4.00%)
Anti-LKM1		Negative < 1/40		46 (92.00%)
		1/80		2 (4.00%)
		> 1/80		2 (4.00%)
AMA		Negative		45 (90.00%)
		Positive		5 (10.00%)
Type of AIH		Type I		41 (82.00%)
		Type II		5 (10.00%)
		Overlap		4 (8.00%)
Liver biopsy	Degree of HAI	Minimal (1–3)		7 (14.00%)
		Mild (4–8)		20 (40.00%)
		Moderate (9–12)		16 (32.00%)
		Severe (13–18)		7 (14.00%)
	Degree of fibrosis	No (F0)		0 (0.00%)
		Mild fibrosis (F1)		6 (12.00%)
		Mild to moderate fibrosis (F2)		6 (12.00%)
		Moderate fibrosis (F3)		15 (30.00%)
		Moderate to severe fibrosis (F4)		16 (32.00%)
		Incomplete cirrhosis (F5)		6 (12.00%)
		Cirrhosis (F6)		1 (2.00%)
	Cells	Eosinophils		5 (10.00%)
		Lymphocytes		10 (20.00%)
		Plasma cells		30 (60.00%)
		Plasma cells & Esinophils		4 (8.00%)
		Plasma cells & Neutrophils		1 (2.00%)
	HAI score/18			8.80 ± 2.80
	Fibrosis index/6			3.10 ± 1.40
Diagnostic scores, treatment regimen and response	Simplified Scoring System			6.90 ± 0.70
	Revised Original Scoring System			22.10 ± 3.20
	Child Pugh score			7.90 ± 1.60
	PELD			8.50 ± 3.50
	MELD			9.40 ± 3.20
	APRI score			5 ± 3.10
	FIB-4 score			0.90 ± 0.30
	Treatment regimen		Steroids	24 (48.00%)
			Steroids & azathioprine	26 (52.00%)
	Treatment response		Relapse	21 (42.00%)
			Remission	29 (58.00%)

Data are presented as mean ± SD or frequency (%). *ANA*, antinuclear antibodies; *ASMA,* anti-smooth muscle antibodies; *Anti-LKM1,* anti-liver kidney microsomal type 1 antibody; *AMA,* antimitochondrial antibody; *HAI,* histological activity, index; *PELD,* pediatric end-stage liver disease; *MELD,* model for end-stage liver disease; *APRI,* AST to platelet ratio index; *FIB-4,* fibrosis-4 index; *AIH,* autoimmune

Elevated GP-73 levels were observed in patients presenting with pallor, jaundice, abdominal pain, abdominal enlargement, ascites, and organomegaly. No significant difference in GP-73 levels was found based on sex. GP-73 levels increased with higher grades of HAI and advanced fibrosis stages, while no significant differences were detected with respect to cell type, AIH subtype, treatment regimen, or treatment response **(**Table 4**).**

**Table 4:** Serum GP-73 levels in relation to clinical features, histological findings, treatment regimens, and treatment response in AIH Patients

			GP-73 (ng/L)	Test	*P* value
Clinical criteria	Sex	Male	111.00 ± 27.40	U = 0.25	0.80
		Female	110.50 ± 26.20		
	Pallor		117.90 ± 30.70	U = 4.40	< 0.001*
	Jaundice		116.10 ± 27.50	U = 3.90	< 0.001*
	Abdominal pain		119.90 ± 27.30	U = 3.20	< 0.001*
	Abdominal enlargement		113.30 ± 29.70	U = 6.30	< 0.001*
	Ascites		111.80 ± 26.00	U = 2.90	0.003*
	Organomegaly	Splenomegaly	107.70 ± 24.20	W = 53.70	< 0.001*
		Hepatomegaly	112.90 ± 30.50		
		HSM	119.40 ± 29.30		
Results of biopsy	Degree of HAI	Minimal	81.10 ± 6.40	W = 32.20	< 0.001*
		Mild	96.10 ± 12.60		
		Moderate	121.60 ± 15.00		
		Severe	157.60 ± 8.70		
	Degree of fibrosis	F1	81.30 ± 7.70	W = 20.10	< 0.001*
		F2	94.20 ± 19.10		
		F3	106.60 ± 19.00		
		F4	109.90 ± 22.80		
		F5	157.60 ± 8.70		
		F6	175.30		
	Cells	Eosinophils	119.20 ± 26.20	W = 5.20	0.38
		Lymphocytes	97.60 ± 20.90		
		Plasma cells	114.70 ± 27.90		
		Plasma cells & Esinop	105.60 ± 33.20		
		Plasma cells & Neutro	115.30		
Type of AIH		Type 1	111.10 ± 17.80	W = 32.20	< 0.001*
		Type 2	106.10 ± 12.40		
		Overlap	107.60 ± 15.00		
Treatment regimen		Steroids	110.80 ± 25.20	U = 0.32	0.76
		Steroids and azathioprine	110.50 ± 28.00		
Treatment response		Relapse	112.60 ± 25.60	U = 0.51	0.60
		Remission	109.40 ± 27.50		

Data are presented as mean ± SD. *U*: Mann–Whitney U test; *W*: Kruskal–Wallis test, *HAI*: Histological Activity Index; *AIH*: autoimmune hepatitis; *HSM*: hepatosplenomegaly,*:Statistically significant at *P* ≤ 0.05

In AIH cases,correlations between GP-73 and clinical parameters showed positive associations with ALT, AST, GGT, ALK.P, bilirubin levels, IgG, coagulation times (PT, PTT, INR), liver span, and spleen size. Negative correlations were observed with hemoglobin, platelet count, and albumin. GP-73 also correlated significantly with age, anthropometric indices, WBCs, creatinine, APRI score, FIB-4 score, HAI score, and degree of fibrosis. No significant correlation was found between GP-73 and other diagnostic criteria of AIH (Table 5).

**Table 5:** Correlation between serum GP-73 levels and clinical, laboratory, and histopathological parameters in children with autoimmune hepatitis

		GP-73 (ng/L)	
		r	*P* value
Clinical data	Age	0.133	0.187
	Weight percentile	−0.209	0.067
	Height percentile	−0.146	0.123
	BMI percentile	−0.214	0.061
	Hb	−0.460	< 0.001*
	WBCs	0.176	0.108
	Platelets	−0.747	< 0.001*
	ALT	0.625	< 0.001*
	AST	0.765	< 0.001*
	GGT	0.287	0.004*
	ALP	0.318	< 0.001*
	Bilirubin total	0.365	< 0.001*
	Bilirubin direct	0.345	< 0.001*
	Albumin	−0.525	< 0.001*
	IgG	0.688	< 0.001*
	Creatinine	0.166	0.098
	aPTT	0.570	< 0.001*
	PT	0.555	< 0.001*
	INR	0.533	< 0.001*
	Liver span	0.450	< 0.001*
	Spleen size	0.522	< 0.001*
Diagnostic criteria of AIH	APRI score	0.861	< 0.001*
	FIB4 score	0.881	< 0.001*
	Simplified scoring system	0.187	0.119
	Revised score	0.272	0.096
	Child Pugh Score	0.062	0.668
	PELD Score	0.319	0.077
	MELD Score	0.284	0.084
	HAI score	0.879	< 0.001*
	Degree of fibrosis	0.647	< 0.001*

Data are presented as correlation coefficient (r),*: Statistically significant at P ≤ 0.05,*BMI,* body mass index; *Hb,* hemoglobin; *WBCs,* white blood cells; *ALT,* alanine aminotransferase; *AST,* aspartate aminotransferase; *GGT,* gamma-glutamyl transferase; *ALP,* alkaline phosphatase; *PT*: prothrombin time; *aPTT,* activated partial thromboplastin time; *INR,* international normalized ratio; *IgG,* immunoglobulin *G,* APRI: AST to Platelet Ratio Index; *FIB-4,* Fibrosis-4 index; *PELD,* Pediatric End-stage Liver Disease score; *MELD,* Model for End-stage Liver Disease score; *HAI,* Histological Activity Index

**Fig. 1:**
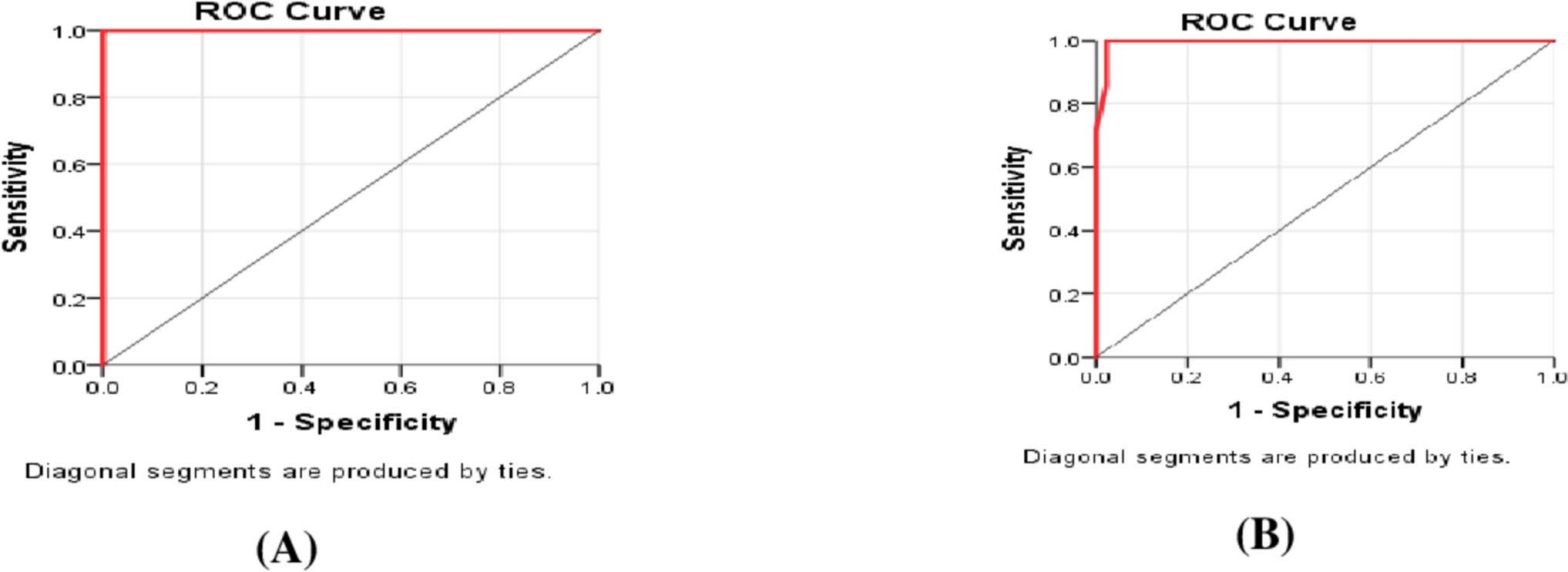
(**A**): ROC curve of performance of GP.73 to detect cases with autoimmune hepatitis, (**B**): To detect cases with severe fibrosis and cirrhosis with AIH

In ROC analysis, GP-73 demonstrated excellent diagnostic accuracy for identifying AIH **(**Fig. 1A**)**, with an AUC of 1.000 (p < 0.001, achieving 100% sensitivity and specificity at a cutoff value > 49 ng/mL. For detecting severe fibrosis **(**Fig. 1B**)** and cirrhosis (F4–F6), the AUC was 0.995 (p < 0.001), with 100% sensitivity and 91.7% specificity at a cutoff > 124.6 ng/mL.

Serum GP-73 concentrations were significantly elevated in patients with autoimmune hepatitis (AIH) compared to healthy controls. The mean level in the AIH group was 110.7 ± 26.5 ng/mL (range: 72–175.3), while the control group had a mean of 21.3 ± 2.5 ng/mL (range: 17–26). The difference betGP-73 the groups was highly significant (U = 8.6, p < 0.001), indicating a strong discriminatory potential for GP-73 in identifying AIH cases (as shown in supplementary Table 1)**.**

The diagnostic performance of GP-73 in distinguishing autoimmune hepatitis (AIH) patients from healthy controls was excellent. Receiver operating characteristic (ROC) analysis revealed an area under the curve (AUC) of 1.000 (95% CI: 1.0–1.0, p < 0.001). At a cutoff value of > 49 ng/mL, GP-73 demonstrated perfect diagnostic accuracy, achieving 100% sensitivity and 100% specificity, as well as 100% positive and negative predictive values (as shown in supplementary Table 2).

Regarding the identification of AIH patients with severe fibrosis and cirrhosis (F4–F6), GP-73 maintained near-perfect diagnostic performance. The AUC was 0.995 (95% CI: 0.981–1.000, p < 0.001), with a sensitivity of 100% and a specificity of 91.7% at a cutoff level > 124.6 ng/mL. Positive and negative predictive values were 90.4% and 100%, respectively (as shown in supplementary Table 3).

## Discussion

An inflammatory liver disease mediated by the immune system, the exact origin of which is still a mystery, AIH can strike individuals of any age, gender, or ethnicity. It should be considered a diagnosis for all patients with acute or chronic inflammation of the liver, regardless of whether they are symptomatic, chronically sick, or exhibit symptoms of acute liver failure [1].

Our results were in agreement with Fouad et al. [17], who reported that at the time of diagnosis, 46.4% of patients were underweight; among whom 42.9% experienced stunting, and 3.6% wasting. All weight indicators increased significantly at follow-up, reducing the underweight rates to 17.8%, mainly in the stunted group.

Our results were partially consistent with Mogahed et al. [18], who studied 34 Egyptian children with AIH (22 girls, 12 boys; mean age 7.2 ± 2.8 years). Concurrent autoimmune diseases were present in 14.7%. Most patients (79.4%) presented with jaundice, while others had hematoemesis (8.8%), abdominal distention (5.8%), or bleeding tendencies (2.9%). Similarly, this was comparable with Mogahed et al. [18], who found pancytopenia in 3.5%, thrombocytopenia in 10%, and anemia in 8.3% of patients. All had elevated transaminases, and many showed elevated bilirubin (76.4%), GGT (91%), and AP (35.2%). Hypoalbuminemia and coagulopathy were also frequent. ASMA, ANA, anti-LKM1, and AMA were variably positive. Most cases were AIH-1 (70.5%). Our results agreed with Mogahed et al. [18], who found that among 19 biopsied patients, 79% showed piecemeal necrosis and 84.2% distorted hepatic architecture. Biliary involvement was seen in 21%. The Ishak fibrosis scores ranged from 2 to 5, and HAI from 6 to 13. In the study by Mogahed et al. [18], patients were initially prescribed oral prednisolone, and 88.2% received azathioprine in addition. Ursodeoxycholic acid was added for those with elevated biliary enzymes. Twelve patients received UDCA, and eight of them achieved normalization of liver enzymes.

In the present study, patients with AIH had significantly higher ALT, AST, GGT, ALP, total bilirubin, direct bilirubin, PT, PTT, INR, WBCs, total IgG, and significantly lower Hb, platelets, and albumin compared to controls. Additionally, 24% had ascites, 84% hepatomegaly, and 42% splenomegaly, with significantly larger liver span and spleen size.

Our findings aligned with Jiménez-Rivera et al. [19], who reported that AIH patients had significantly lower serum albumin (3.3 vs. 3.8 mg/dL), lower platelet count (187,000 vs. 249,000; p < 0.001), and higher INR (1.4 vs. 1.2; p < 0.001) compared to controls.

In our study, the mean HAI was 8.8 ± 2.8, with most cases being mild (40%), followed by moderate (32%), severe (14%), and minimal (14%). The mean fibrosis score was 3.1 ± 1.4, 12% had mild fibrosis (F1), 12% had mild to moderate fibrosis (F2), 30% had moderate fibrosis (F3), 32% had moderate to severe fibrosis (F4), 12% had incomplete cirrhosis (F5), and 2% had cirrhosis (F6). Plasma cells were the predominant cell type observed in liver biopsies (60%).

Behairy et al. [20], reported findings consistent with our observations regarding liver disease severity in pediatric patients. They classified 28.8% of patients as Child–Pugh class A, 61.2% as class B, and 10% as class C. All patients initially received corticosteroids, and 54 were later treated with azathioprine. Remission was achieved in 57.5% of patients, while 42.5% experienced relapses. These data highlight the variability in disease progression and treatment response, underscoring the need for reliable biomarkers like GP-73 for better disease staging and monitoring in clinical practice.

In the current study, patients with AIH had significantly higher GP-73 compared to controls (110.7 ± 26.5 vs. 21.3 ± 2.5 ng/mL, p < 0.001). To the best of our knowledge, this is the first study to assess the role of GP-73 in AIH in children. GP-73 levels were significantly higher in patients presenting with pallor, jaundice, abdominal pain, ascites, and organomegaly, and in those with higher grades of HAI and fibrosis. No differences were observed based on sex, cell type, or AIH type. GP-73 showed positive correlations with liver function markers and fibrosis scores, and no differences were found according to treatment or response. This supports its utility in disease staging and monitoring treatment response.

The primary objective of this study was to explore the association between serum GP-73 levels and the degree of hepatic fibrosis in children with autoimmune liver disease. Our findings demonstrated that GP-73 levels were significantly elevated in patients with higher stages of fibrosis (F4–F6) and histological activity index (HAI), indicating a strong correlation between GP-73 and disease severity (Table 4). Moreover, GP-73 showed a significant positive correlation with non-invasive fibrosis scores such as APRI and FIB-4 **(**Table 5**)**, supporting its potential utility as a biomarker of liver fibrosis in pediatric AIH.The incorporation of noninvasive tests, such as serum biomarkers and elastography, into the routine evaluation of autoimmune hepatitis offers a promising adjunct to biopsy, especially in pediatric populations where repeated histological assessments are not always feasible [21]. The diagnostic performance was further confirmed by ROC analysis, where GP-73 achieved excellent accuracy in detecting advanced fibrosis, with an AUC of 0.995, sensitivity of 100%, and specificity of 91.7% at a cutoff > 124.6 ng/mL. These findings are consistent with previous studies as Mieli-Vergani et al. [22] who reported that in pediatric autoimmune hepatitis, early identification of severe disease and timely initiation of immunosuppressive therapy are critical to prevent progression to cirrhosis. Non-invasive biomarkers that correlate with histological severity may improve diagnostic accuracy and reduce the reliance on liver biopsy. Thus, our study reinforces the potential role of GP-73 as a non-invasive marker for evaluating liver fibrosis in children. Further longitudinal studies are needed to validate these findings and determine its prognostic value.

Our results are in accordance with those of Yao et al. [7], who first proposed the hypothesis that serum GP-73 could serve as a standalone biomarker for assessing liver necroinflammation. In their study, GP-73 showed AUCs of 0.828 for moderate necroinflammation (≥ G2) and 0.832 for severe necroinflammation (≥ G3), indicating a strong correlation with histological grades. Similarly, in our study, GP-73 demonstrated significant diagnostic performance for necroinflammation, with ROC values of 0.820 (P < 0.001) for moderate necroinflammation and 0.803 (P < 0.001) for severe necroinflammation. Moreover, GP-73 exhibited excellent accuracy in diagnosing autoimmune hepatitis, with an AUC of 1.0, 100% sensitivity and specificity. It also showed high diagnostic value for severe fibrosis and cirrhosis, with an AUC of 0.995, 100% sensitivity, and 91.7% specificity at a cutoff value of > 124.6 ng/mL**.** These findings support the use of GP-73 as a non-invasive biomarker for staging fibrosis and monitoring treatment response in pediatric AIH. Further longitudinal studies are needed to validate its prognostic role.

GP-73 is known to be highly expressed in the epithelial cells of bile ducts, which are primarily located in the portal areas of the liver. In overlap syndromes, such as AIH-PBC or AIH-PSC, these bile ducts are often affected, which may influence GP-73 levels differently than in isolated AIH. In our study, however, no statistically significant difference in GP-73 levels was observed between patients with overlap syndrome and those with type 1 or type 2 AIH **(**Table 4**).**The limited number of overlap cases may have reduced the power to detect a true difference. Nevertheless, this observation aligns with previous studies of yao et al. [7] who reporting elevated GP-73 in cholestatic liver diseases such as primary sclerosing cholangitis and primary biliary cholangitis. Further research involving larger overlap cohorts is needed to better elucidate the role of GP-73 in this subgroup.

Despite the strengths of our study, limitations must be acknowledged. The small sample size and single-center nature may limit generalizability. The cross-sectional design does not allow assessment of dynamic changes or prognostic utility.

Future studies should include multicenter, larger cohorts to confirm findings. Longitudinal designs are essential to assess temporal changes in GP-73 and their relation to disease progression and outcomes. Investigating GP-73’s molecular pathways may also deepen understanding of its role in liver disease.

## Conclusion

Serum GP-73 levels were significantly elevated in children with autoimmune hepatitis compared to healthy controls, with a mean of 110.7 ng/mL versus 21.3 ng/mL. GP-73 levels showed a strong positive correlation with histological fibrosis stages and non-invasive fibrosis scores (APRI, FIB-4), and achieved excellent diagnostic accuracy in detecting severe fibrosis (AUC = 0.995; sensitivity = 100%; specificity = 91.7%). These findings are in line with recent proteomic studies that highlighted GP-73 as a promising non-invasive biomarker for autoimmune liver diseases in children, demonstrating its diagnostic potential in differentiating disease severity. Thus, to validate these findings, additional prospective studies with longer time horizons are necessary. Studies using larger sample sizes and combining GP-73 with other biomarkers may further improve its utility as a marker to identify severe cases of AIH.

## Supplementary Information

Below is the link to the electronic supplementary material.

ESM 1 (PDF 34.1 KB)

ESM 2 (PDF 775 KB)

## Data Availability

No datasets were generated or analysed during the current study.

## Abbreviations

AIH: Autoimmune hepatitis
AILD: Autoimmune liver disease
ALT: Alanine aminotransferase
AST: Aspartate aminotransferase
APRI: Aspartate aminotransferase to platelet ratio index
AUC: Area under the curve
BMI: Body mass index
ELISA: Enzyme-linked immunosorbent assay
FIB-4: Fibrosis-4 score
GP-73: Golgi protein 73
HAI: Histological activity index
HBV: Hepatitis B virus
HCV: Hepatitis C virus
HSM: Hepatosplenomegaly
INR: International normalized ratio
LKM-1: Liver-kidney microsomal antibody type 1
MELD: Model for end-stage liver disease
PELD: Pediatric end-stage liver disease
PT: Prothrombin time
APTT: Activated partial thromboplastin time
WBCs: White blood cells
ASMA: Anti-smooth muscle antibodies
AMA: Antimitochondrial antibodies
