# Supplementary material for: Serum golgi protein-73 (GP-73) in children with autoimmune hepatitis
Source: Eur J Pediatr. 2025 Sep 3;184(9):592. doi: 10.1007/s00431-025-06428-7 (PMC12408767; doi:10.1007/s00431-025-06428-7)
Supplement: Supplementary file 1 — (PDF 34.1 KB) [file 431_2025_6428_MOESM1_ESM.pdf]

Supplementary table 1: Comparison of Serum GP-73 Levels between AIH Patients and Controls.

		AIH group		Control group		Test	P value
		**N=50**	**%**	**N=50**	**%**		
**GP.73 (ng/L)**	**Mean ±SD**	110.70±26.50		21.30±2.50		U=8.60	<0.001*
	**Range**	72-175.30		17-26			

Supplementary table 1: U: Mann-Whitney U-test, *: significant, GP.73: Golgi Protein 73. This table showed that patients with AIH had statistically higher GP.73 compared to controls.

Supplementary table 2: Performance of GP.73 to detect cases with autoimmune hepatitis from controls.

	AUC	95% CI		Cut-off value	Sensitivity	Specificity	PPV	NPV	P value
**GP.73 (ng/L)**	1	1	1	>49	100%	100%	100%	100%	<0.001*

Supplementary table 2: ROC analysis was done to assess the performance of GP.73 to detect cases with autoimmune hepatitis; AUC was 1, p<0.001. At a cutoff point >49 ng/L, the sensitivity was 100% and specificity was 100%.

Supplementary table 3: Performance of GP.73 to detect cases with severe fibrosis and cirrhosis with AIH(F4-F6).

	AUC	95% CI		Cut-off value	Sensitivity	Specificity	PPV	NPV	P value
**GP.73 (ng/L)**	0.995	0.981	1	>124.6	100 %	91.70 %	90.40 %	100%	<0.001*

Supplementary table 3: ROC analysis was done to assess the performance of GP.73 to detect AIH cases with severe fibrosis and cirrhosis; AUC was 0.995, p<0.001. At a cutoff point >124.6 ng/L, the sensitivity was 100% and specificity was 91.7%.
