# Supplementary material for: Serum golgi protein-73 (GP-73) in children with autoimmune hepatitis
Source: Eur J Pediatr. 2025 Sep 3;184(9):592. doi: 10.1007/s00431-025-06428-7 (PMC12408767; doi:10.1007/s00431-025-06428-7)
Supplement: Supplementary file 2 — (PDF 775 KB) [file 431_2025_6428_MOESM2_ESM.pdf]

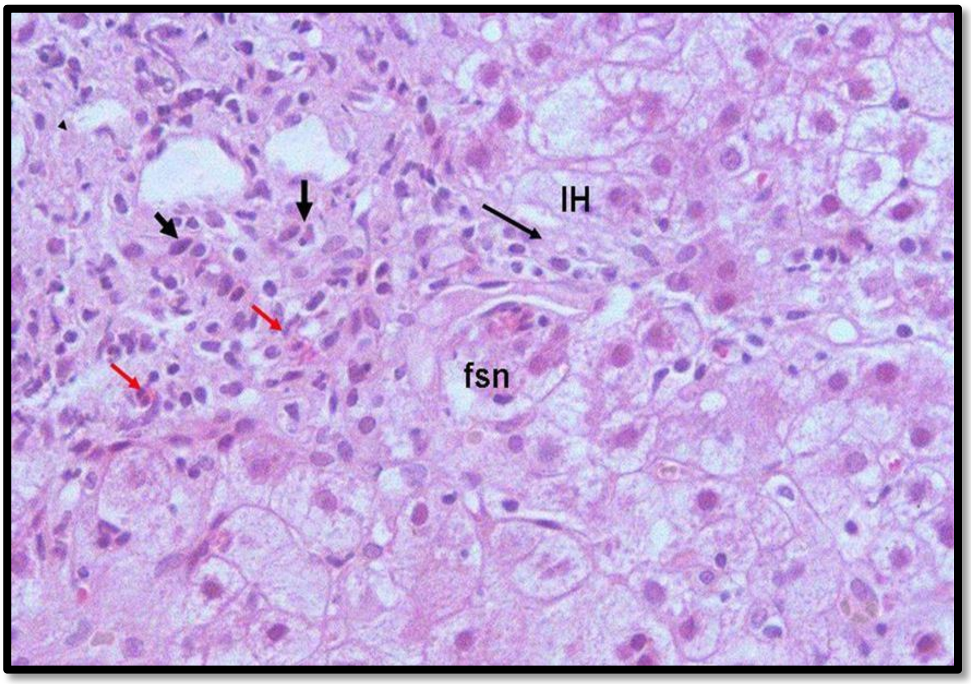

Supplementary Figure 1: Autoimmune hepatitis showing interface hepatitis (IH), focal spoty necrosis (Fsn), mononuclear inflammatory cell infiltrate with numerous plasma cells (short black arrow) and esinophils (red arrow) (H&E X 400).

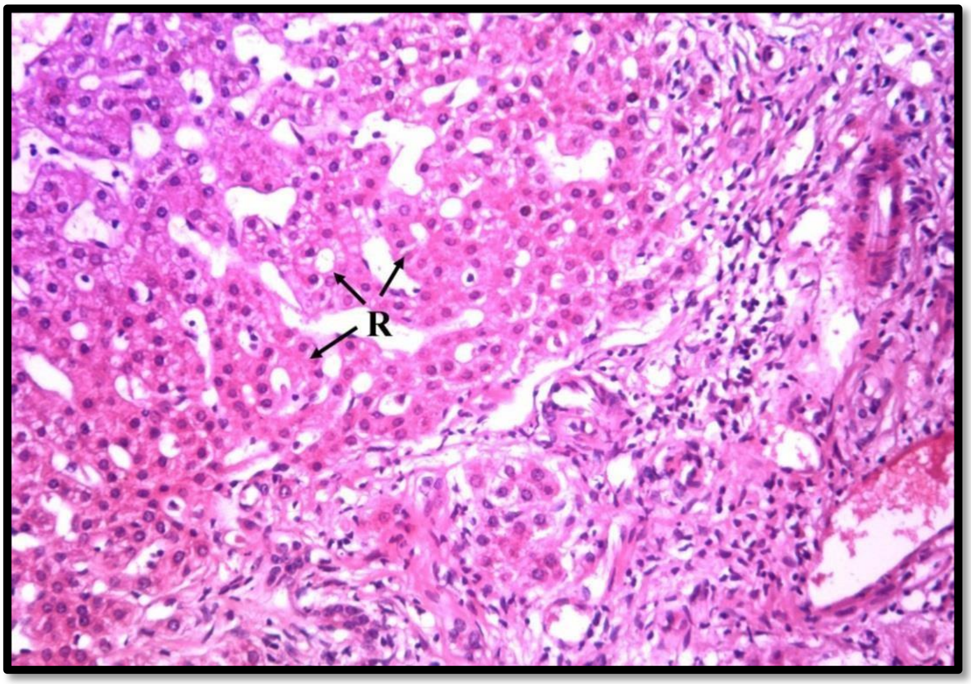

Supplementary Figure 2: Autoimmune hepatitis showing rossete formation of hepatocytes (R) (H&E X200).

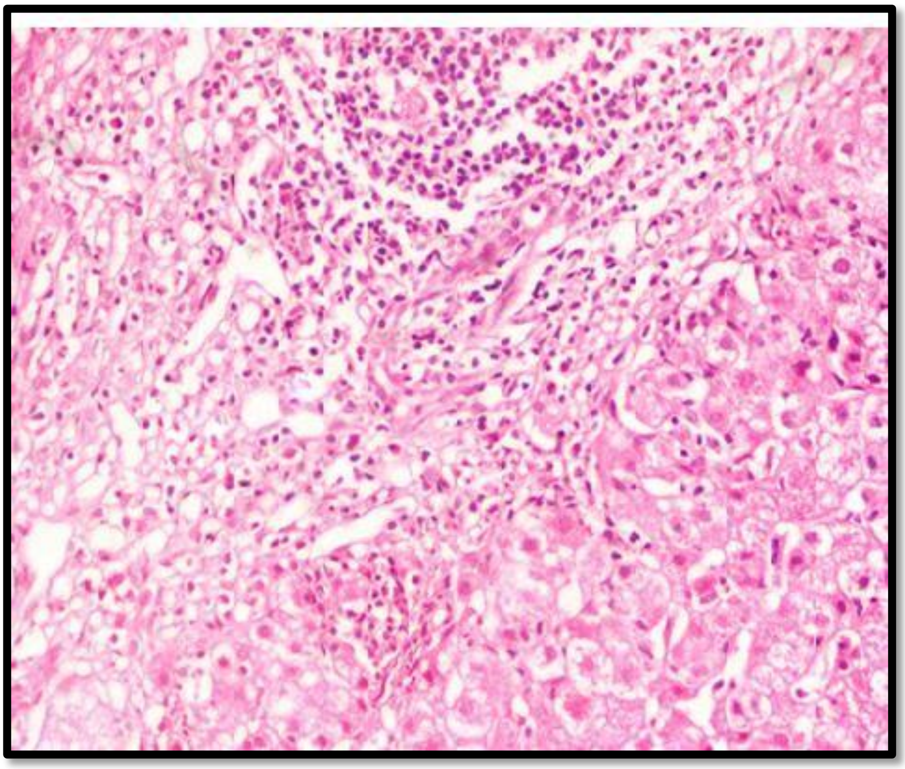

Supplementary Figure 3: Photomicrogragh of H & E stained liver tissue in a case of AIH showing mild activity in the form of mild portal inflammation and focal interface hepatitis (20X HPF)

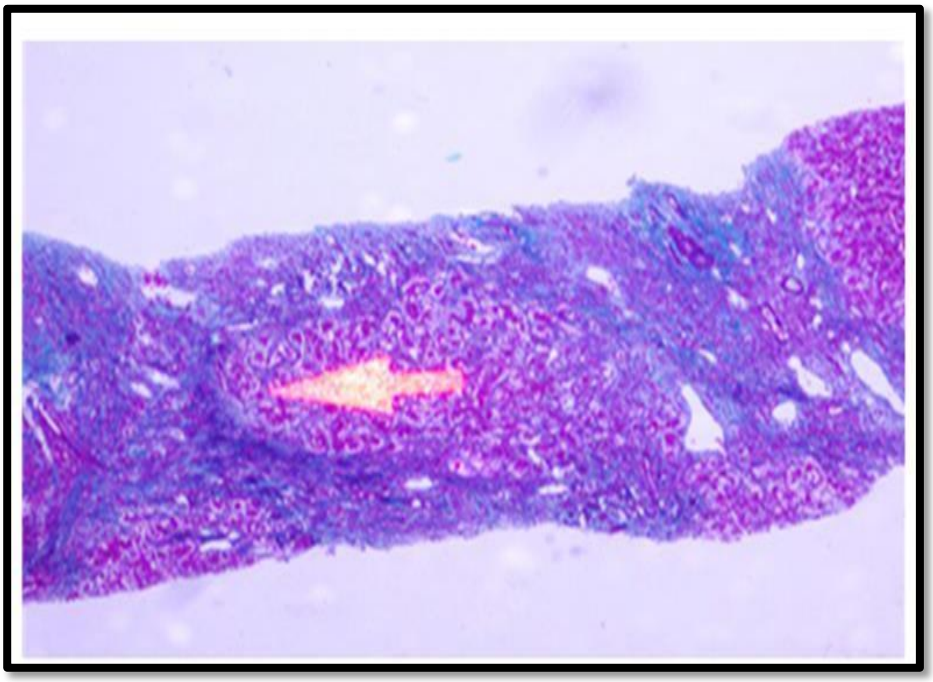

Supplementary Figure 4: Liver tissue in case of AIH stained with masson Trichrome stain and showing severe bridging fibrosis with incomplete nodule formation (red arrow) (4xHPE).

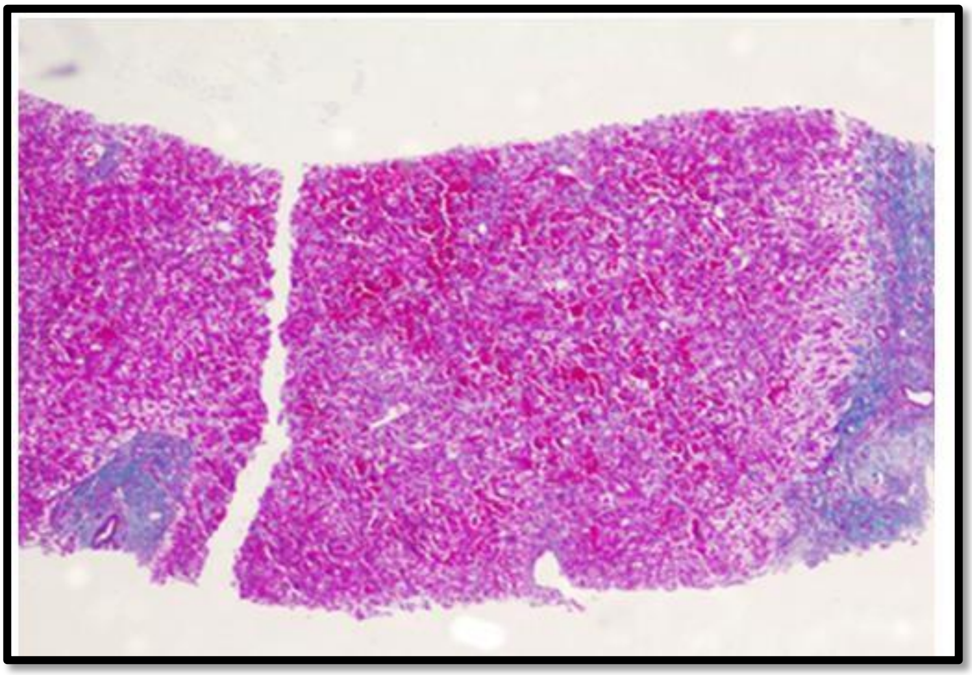

Supplementary Figure 5: Liver tissue in a case of AIH stained with Masson Trichrome stain showing mild portal fibrosis without fibrous links or septa formation (4x HPF).

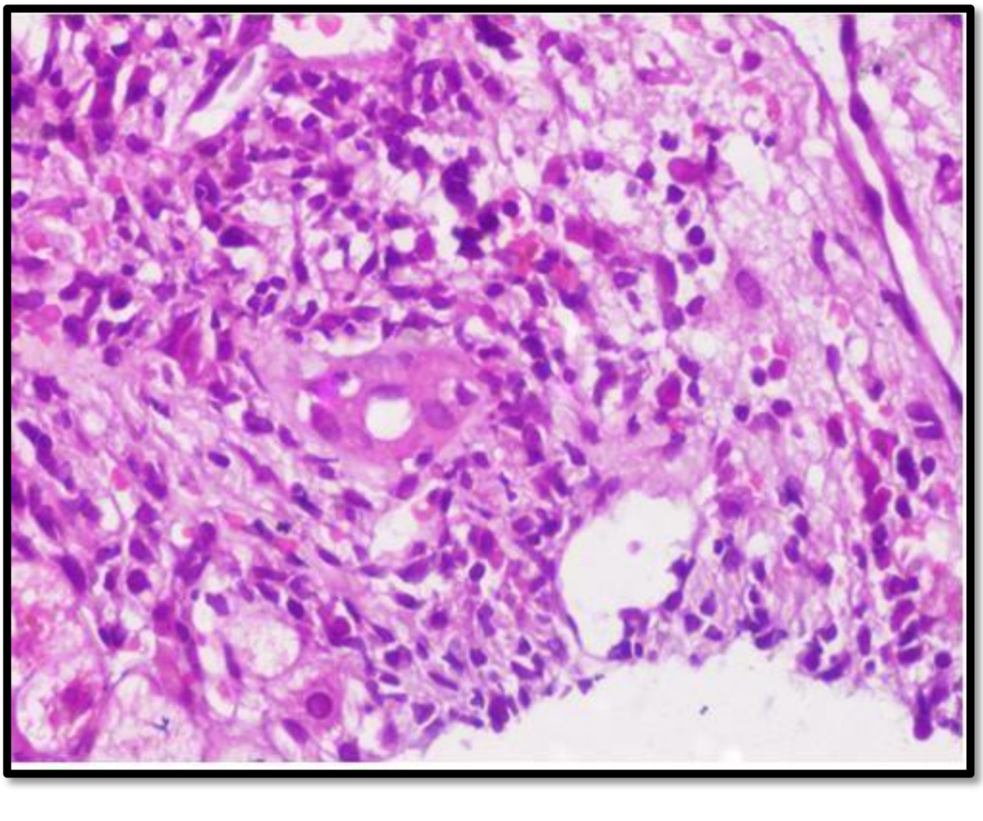

Supplementary Figure 6: Photomicrograph of H & E stained liver tissue in a case of AIH showing mixed inflammatory cells containing lymphocytes, plasma cells and esinophils (40 x HPF).

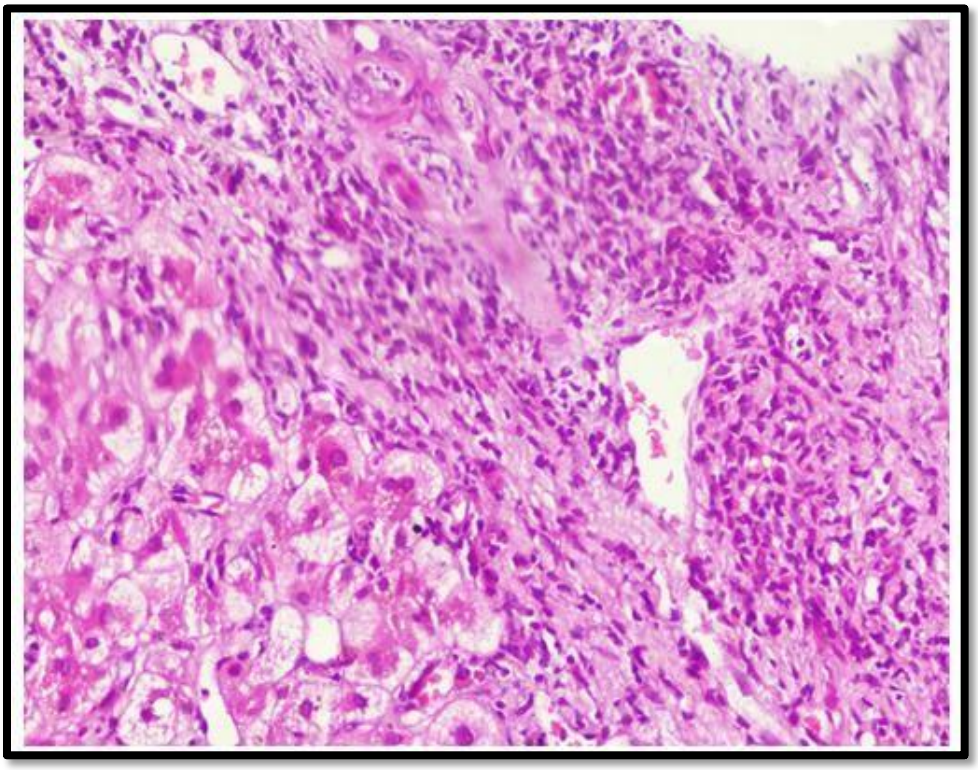

Supplementary Figure 7: Photomicrograph of H & E stained liver tissue in a case of AIH showing severe activity in the form of marked mixed portal inflammation and moderat/marked interface hepatitis (20 X HPE).

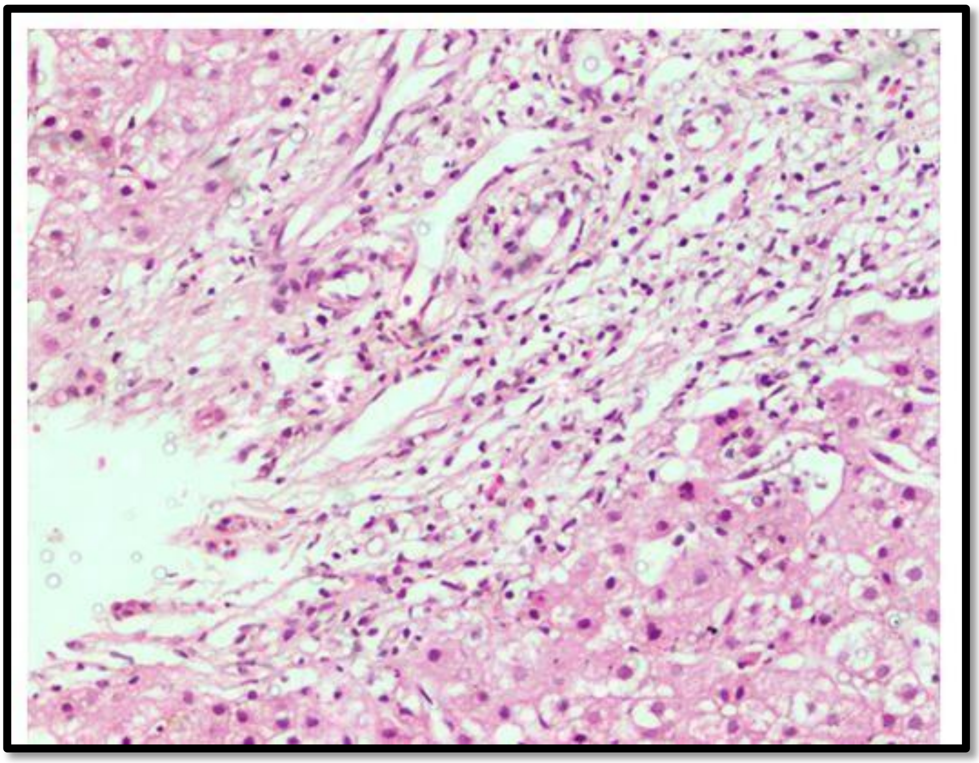

Supplementary Figure 8: Photomicrograph of H & E stained liver tissue in a case of AIH showing moderate activity in the form of moderate portal inflammation and mild / moderate interface hepatitis (20 X HPF).
